# Concerted Gene Expression of Hippocampal Steroid Receptors during Spatial Learning in Male Wistar Rats: A Correlation Analysis

**DOI:** 10.3389/fnbeh.2016.00094

**Published:** 2016-05-17

**Authors:** Gert Lubec, Volker Korz

**Affiliations:** ^1^Department of Pharmaceutical Chemistry, Faculty of Life Sciences, University of ViennaVienna, Austria; ^2^Department of Pediatrics, Medical University of ViennaVienna, Austria

**Keywords:** corticosterone, receptor interaction, estrogen, androgen, spatial learning, memory

## Abstract

Adrenal and gonadal steroid receptor activities are significantly involved and interact in the regulation of learning, memory and stress. Thus, a coordinated expression of steroid receptor genes during a learning task can be expected. Although coexpression of steroid receptors in response to behavioral tasks has been reported the correlative connection is unclear. According to the inverted U-shape model of the impact of stress upon learning and memory we hypothesized that glucocorticoid (GR) receptor expression should be correlated to corticosterone levels in a linear or higher order manner. Other cognition modulating steroid receptors like estrogen receptors (ER) should be correlated to GR receptors in a quadratic manner, which describes a parabola and thus a U-shaped connection. Therefore, we performed a correlational meta-analyis of data of a previous study (Meyer and Korz, 2013a) of steroid receptor gene expressions during spatial learning, which provides a sufficient data basis in order to perform such correlational connections. In that study male rats of different ages were trained in a spatial holeboard or remained untrained and the hippocampal gene expression of different steroid receptors as well as serum corticosterone levels were measured. Expressions of mineralocorticoid (MR) and GR receptors were positively and linearly correlated with blood serum corticosterone levels in spatially trained but not in untrained animals. Training induced a cubic (best fit) relationship between mRNA levels of estrogen receptor α (ERα) and androgen receptor (AR) with MR mRNA. GR gene expression was linearly correlated with MR expression under both conditions. ERα m RNA levels were negatively and linearily and MR and GR gene expressions were cubicely correlated with reference memory errors (RME). Due to only three age classes correlations with age could not be performed. The findings support the U-shape theory of steroid receptor interaction, however the cubic fit suggest a more complex situation, which mechanisms may be revealed in further studies.

## Introduction

Steroids exert significant functions in human and mammalian brains, acting on neuronal and synaptic plasticity and neurogenesis. Through these functions, they are significantly involved in the regulation of stress effects, mood, learning, and memory generation and storage under normal conditions as well as in the development of psychiatric diseases.

A long-standing model to understand the relation between cognitive performance and stress is the inverted-U-shape-hypothesis, which propose better learning and memory during states of intermediate stress (Akirav et al., 2004), whereas very low and escalated stress impairs cognition and motivation in animals and humans (Anderson, 1976; Sandi et al., 1997; Andreano and Cahill, 2006; Salehi et al., 2010). Evidence from behavioral, neuronal and genetic studies support this hypothesis (Diamond et al., 1992; Luksys et al., 2009).

The release of glucocorticoids (GRs) is a valid correlate of stress and has been used to associate stress and behavioral performance (McGaugh, 1983; Akirav et al., 2001; Korte, 2001; Joëls, 2006; Salehi et al., 2010; Schwabe et al., 2010). GRs in the brain act via two distinct receptor populations on neuronal processes: the GR and the mineralocorticoid (MR) receptor, the latter less abundant but with a higher affinity to the ligand. During the last two decades it turned out that probably not only these receptors, sharing the same ligand, but also other steroid receptors interact with GR and MR and among each other. Androgen (AR) and estrogen receptors (ER), which regulation has been described to be stress related, can communicate with GR receptors by means of heterodimerization (Chen et al., 1997; Cvoro et al., 2011). Estrogen receptor α (ERα)/ERβ heterodimers (Chen et al., 1997; Savatier et al., 2010), interact with GR receptors (Cvoro et al., 2011) causing mutual enhancement or reduction of related target gene expression.

Recently, estrogenic functions in males have been reorted (Gagnidze and Pfaff, 2009; Wu et al., 2009), the ligand provided by local conversion of testosterone into estradiol by the enzyme aromatase. ERα and β are expressed particularly in the hippocampus in males (Weiland et al., 1997; McEwen, 2002; Kalita et al., 2005), regulating spinal plasticity and long-term potentiation (LTP; Day et al., 2005; Liu et al., 2008; Kramár et al., 2009), as well as spatial learning (Frye et al., 2007; Liu et al., 2008; Rissman, 2008; Neese et al., 2010). However, the effects are contradictory depending on the behavioral task (Tetel and Pfaff, 2010).

This task specificity together with the interaction between steroid receptor makes it feasible that the expression of steroid receptors is regulated in a concerted mannner in response to the task (McEwen, 1992; van Steensel et al., 1996; Oitzl et al., 1997). Mahfouz et al. (2016) observed coexpressions of six nuclear steroid receptors in male mice including the here studied receptors even in different brain regions suggesting a coordinated regulation of those regions by GRs and estrogens. The possible systematic relationships between different receptor expressions however have not been described so far. According to the inverted U-shape hypothesis regarding stress and memory different steroid receptors should be coexpressed in a quadratic manner. For instance, plasticity and cognition supporting ER should be maximally coexpressed with GR receptors (regulating the stress response) when the latter are at an intermediate level. Using a meta-analytic correlational approach of data of an earlier study (Meyer and Korz, 2013a), we tried to figure out whether such relations in the coexpression of different steroid receptor genes could be found. Therefore, we compared coexpressions under learning and non-learning conditions in male rats by linear and nonlinear regression, and their relations to individual memories. Meta-analyses of previous data in a first step avoid sacrificing a new and large cohort of animals. The results however give valuable hints that further studies may be useful in enhancing the understanding of steroid receptor network mechanisms.

## Materials and Methods

Methodological details about housing, spatial training, hormone assaying and quantitative real time RT-polymerase chain reaction are given in Meyer and Korz (2013a).

Male Wistar rats at an age of 8 to 24 weeks were used. The animals underwent spatial training by use of always the same holeboard protocol or remained untrained. All animals (trained and untrained) were food deprived and i.c.v cannulated but did not receive a pharmacologically effective treatment. The control groups stayed within the testing room throughout the experiment. All animals were sacrificed at the same time point (15 min after the retention trial, i.e., between 10:15 and 10:30 a.m.). Raw data were taken from Meyer and Korz (2013a), and were differently analyzed in the present study.

### Statistical Analyses

All statistical analyses were made by SPSS (V. 18). Regression curve fit analyses. Linear, quadratic and cubic regression algorithms have been tested. The quadratic function (Y’ = a + b_1_X_1_ + b_2_X_1_^2^) is a second order polynomial regression representing the inverted U-shape model describing a parabola. The cubic regression is a third order polynomial regression similarily shaped (Y’ = a + b_1_X_1_ + b_2_X_1_^2^ + b_3_X_1_^3^). Higher order regressions have not been tested. Regressions coefficients were considered only if the regression analysis of variance (ANOVA) table was significant, meaning that the *p*-value is below 0.05 indicating that the curve fits (least square) the data. The model which explained most of the variance is given in the figures. Deviation from the model was tested by using the Wald-Wolfowitz runs test. Differences in slopes or intercepts were tested by analysis of covariance (ANCOVA). All groups described in the Meyer and Korz (2013a) study has been included in this study. However correlational studies require complete hormone and receptor data sets of individual rats. Because corticosterone concentrations were not measured for all animals from which the molecular measures were taken, receptor and hormone regression analyses cover lower sample sizes as the receptor-receptor analyses, not for all animals where corticosterone has been measured all receptor RNAs were available and sample sizes in receptor RNA correlations vary because not for each animal RNAs for all receptors could be measured. Corticosterone concentration comparisons between groups were done by the Student’s *t*-test. The level of significance for all tests was set at *p* ≤ 0.05 (two-tailed).

## Results

### Correlations of Body Weights with Corticosterone

We did not found any correlations with body weights, nor in the percentages of decrease due to the food deprivation and neither in the total body weights. Blood serum corticosterone concentrations were not different in trained (214.2 ± 50.3 ng/ml, *n* = 10) and untrained (180.7 ± 24.6 ng/ml, *n* = 18, *t* = −0.67, *p* > 0.1) animals.

### Correlations of Steroid Receptor mRNA with Corticosterone

**Figure 1:**
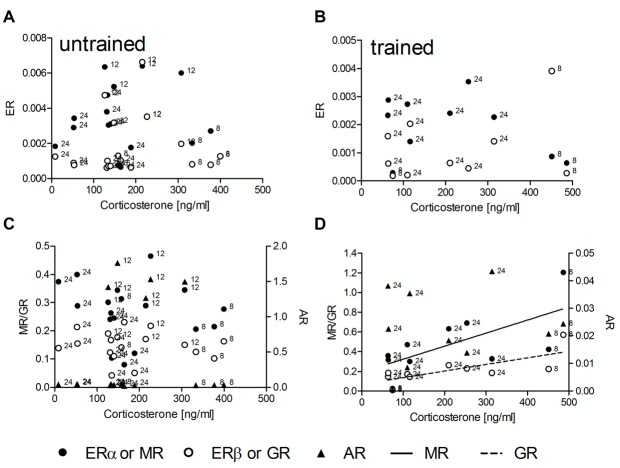
**The hippocampal relative expression of glucocorticoid (GR) and mineralocorticoid (MR) mRNA levels are correlated with serum corticosterone concentrations only in trained (D) but not in untrained (C) male rats.** Estrogen receptor α (ERα) and ERβ mRNA **(A,B)** as well as androgen receptor (AR) mRNA **(C,D)** levels are uncorrelated with serum corticosterone under both conditions. MR and GR data are plotted against the left ordinate and the AR data against the right ordinate in **(C,D)**. Given are the relative gene expressions. The number at the left side of each data point give the age (in weeks) of the animal from which the sample is taken.

In untrained animals (*n* = 18) no linear or non-linear correlations of corticosterone with the expression levels of mRNA for either ER, MR or GR could be determined (Figures 1A,C). Also trained animals (*n* = 10) did not show any correlation between hormone concentrations and mRNA levels for ERα, ERβ and AR (Figures 1B,D). Significant linear correlations of corticosterone concentrations with levels of receptor mRNA were found only in trained animals with MR (*R^2^* = 0.44, *p* = 0.036) and GR (*R^2^* = 0.55, *p* = 0.015) mRNA levels. The runs tests revealed no deviation from linearity: *p* = 0.881 and *p* = 0.833; respectively. While the slopes of regression lines are not different (*F* = 1.35; DF_(1,17)_; *p* = 0.262) the intercepts are different (*F* = 9.23; DF_(1,17)_; *p* = 0.007).

### Correlations Between Different Steroid Receptor mRNA Levels

**Figure 2:**
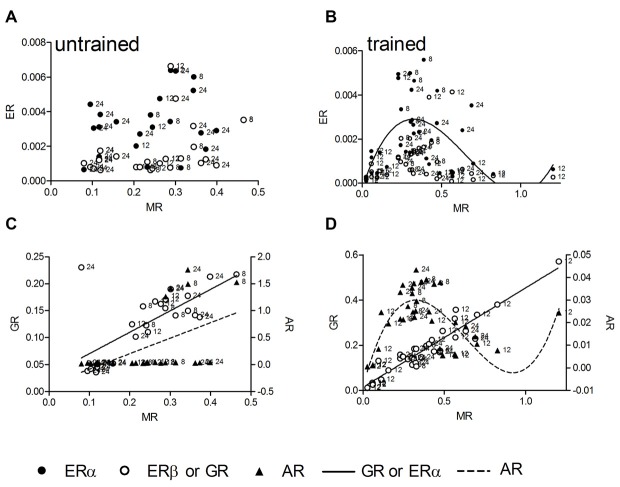
**The hippocampal relative expression of ERα and ERβ mRNA gene expressions are not correlated with hippocampal MR mRNA levels in untrained animals (A).** In trained animals a significant cubic regression for ERα and a trend (*p* = 0.069) for ERβ receptor mRNA with MR mRNA can be observed **(B)**. The AR and GR mRNA are linearly correlated with MR mRNA in untrained animals **(C)**, in trained animals the linear correlation of GR mRNA persists **(D)**, whereas the AR mRNA is correlated in a cubic manner with MR mRNA **(D)**. MR and GR data are plotted against the left ordinate and the AR data against the right ordinate in **(C,D)**. Given are the relative gene expressions. The number at the left side of each data point give the age (in weeks) of the animal from which the sample is taken.

We tested linear and nonlinear functions of mRNA expression with MR as independent variable. MR mRNA is linearly correlated with corticosterone, thus mRNA levels reflect stress levels, and in contrast to GR, has been identified as crucial receptor to be involved in spatial long-term memory as well as LTP during the standard holeboard protocol (Korz and Frey, 2007) that has been used also in this study. In untrained animals (*n* = 25) there was no correlation between MR and ERα as well as ERβ (Figure 2A), whereas a linear correlation of MR mRNA with that of GR (*R^2^* = 0.49, *p* < 0.001) and AR (*R^2^* = 0.26, *p* = 0.008) could be determined (Figure 2C). Slopes of the GR and AR regression lines are significantly different (*F* = 6.13; DF_(1,46)_; *p* = 0.017) The MR-GR correlation is not deviated from linearity (*p* = 0.415), whereas the MR-AR regression deviates from linearity (*p* = 0.002). Thus, the latter model mostly depend on only a few animals showing elevated AR expression with increased MR mRNA. The low portion of variance of only 26% explained by the linear function also points to the weakness of the relation. In trained animals (*n* = 37, Figures 2B,D) however, a complete different situation appears. For the ERα and AR mRNA levels a cubic correlation with MR expression explains the highest portion of variance among variables (ERα: *R^2^* = 0.32, *p* = 0.008; Figure 2B), (AR: *R^2^* = 0.67, *p* < 0.001; Figure 2D). There is no deviation from the model in ERα (*p* = 0.886) as well as in AR (*p* = 0.844). The GR mRNA concentrations again show a linear correlation with MR expression (*R^2^* = 0.90, *p* < 0.001), showing no deviation from linearity (*p* = 0.442). Cubic regressions in all cases are not dependent on the points of highest MR expression, but remained significant if these points are excluded from the analyses.

### Correlations Between Steroid Receptor mRNA Levels and Behavior

**Figure 3:**
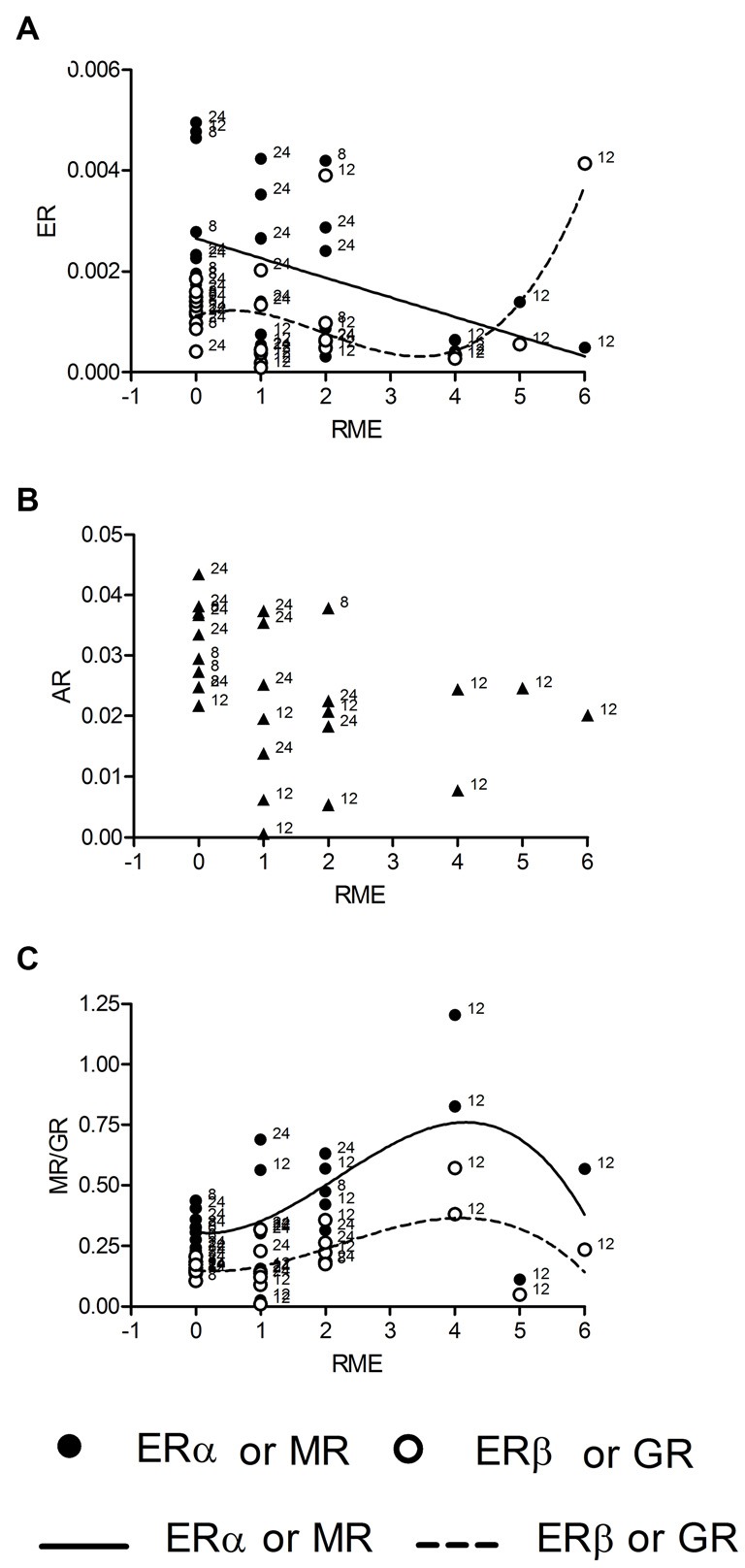
**ERα mRNA is linearly and ERβ mRNA cubically (A) correlated with the numbers of reference memory errors (RME).** Both MR and GR mRNAs are correlated with RME in a cubic manner **(C)**, whereas AR mRNA is not correlated with RME **(B)**. Given are the relative gene expressions. The behavioral data are taken from the retention trial (trial 10, day 3). The number at the left side of each data point give the age of the animal from which the sample is taken.

We also tested correlations for mRNA concentrations with of reference memory errors (RME) made by individual rats during the last trial of the holeboard training. We found correlations between RME and all receptor expressions except AR (Figure 3B). The regression of mRNA levels and RME was linear for ERα (*R^2^* = 0.19, *p* = 0.027; Figure 3A, *n* = 26), fitting to the model (*p* = 0.735) and cubic for ERβ (*R^2^* = 0.33, *p* = 0.038, Figure 3A, *n* = 26), GR (*R^2^* = 0.34, *p* = 0.032, Figure 3C, *n* = 25) and MR mRNA (*R^2^* = 0.33, *p* = 0.037; Figure 3C, *n* = 26). However ERβ and MR correlations deviates from the model (both *p* = 0.036), whereas the GR correlation fits into the model (*p* = 0.207). There was no correlation of corticosterone titers with RME.

## Discussion

According to the inverted U-shape model of the impact of stress upon learning and memory we hypothesized that GR receptor expression should be correlated to corticosterone levels in a linear or higher order manner. Other cognition modulating steroid receptors like ER should be correlated to GR receptors in a quadratic manner, which describes a parabola and thus a U-shaped connection. GR receptore are lineary correlated with serum corticosterone levels only in trained animals but not untrained animals although the hormone levels are not different between these groups. Thus, in trained animals the expression of MR and GR reflects the individual amount of circulating corticosterone. The relatively high concentrations of serum corticosterone in untrained animals is well known as an outcome of food deprivation, that induces chronically elevated serum corticosterone concentrations (Krieger, 1974; Itoh et al., 1980; Schulz and Korz, 2010), the variation may be due to the individual sensitivity to less food. Hippocampal corticosterone concentrations lack to correlate with the receptor levels, however this may be a matter of timing, because serum concentrations react very quickly to environmental demands and it takes some time to see this changes also in the hippocampus, especially at the individual level. ERα, ERβ and AR expression seems to be independent of corticosterone under both environmental conditions but not independent of MR expression after training. According to the hypothesis the relationship between MR and ER should be best described by a quadratic function. However, a cubic regression function thus a third order regression, explaining most of the variance, fits best to the data. There is some support of the initial hypothesis, however the relationship seems to be more complicated. Although, we cannot make any conclusions on causal effects based on the present analyses, these results suggest that the inverted U-shape (quadratic) relation is reflected in mRNA expression only during intermediate to higher stress, but the regulation of gene expression may involve other factors when stress or GRs reach a critical level. The identification of these factors and their possible involvement in stress related cognitive decline may be a promising field of research in future studies. The possibly changing relative occupation of MR and GR under these conditions may be one factor involved.

It has been suggested that MR act as a molecular switch to direct downstream signaling processes involved in spatial navigation (Oitzl and de Kloet, 1992; Schulz and Korz, 2010) and memory systems as well as in the maintenance of hippocampal LTP (Wang et al., 2013), a cellular model of memory formation. The balance of MR/GR and the relative occupation plays a major role on stress related cognition and motivation. Some previous studies addressed specific functions of GR receptors using genetic mouse models. Mice with genetically reduced hippocampal GR expression exhibit depression like behavior whereas GR overexpression reduced helplessness after stress (Ridder et al., 2005). Reduced forebrain GR levels result in enhanced whereas increased MR at the same time reduced the hypothalamo-pituitary-adrenal (HPA)-axis activity, enhanced spatial perseverance and impaired fear memory extinction (Harris et al., 2013). Parallelism in MR and GR gene expression can be observed under both environmental conditions, thus seems to require the combination of stress and training, as suggested by the lack of correlations with corticosterone levels in untrained animals. AR expression is related to MR expression in a similar manner as ERα in trained animals, possibly suggesting similar functional relations of these receptors with MR induced cellular signaling or expression of target genes of MR during training. Target genes of activated receptors may be involved in the control of cognition and emotion. The whole network then may act not only to promote but also to reduce the emotional-cognitive processing, thus keeping the system in a homeostasis. Two main preliminary conclusions can be drawn from these results: (i) a learning related quadratic or cubic function of single steroid receptors cannot be observed, but can be realized by the interplay of different receptor types; and (ii) the kind of correlation between expressions of specific steroid receptors can be different when related to memory. The second point has been confirmed with regard to corticosterone effects (downstream signaling proteins and local availability of corticosterone) in different brain regions (Akirav et al., 2001; Joëls, 2006).

The interaction of different receptors take place by different mechanisms. Heterodimerization, the combination of different receptor types in heterodimeric protein complexes, changes the availability of target gene promoter binding sites and thus the expression of target genes. Heterodimerization and other protein-protein interactions of steroid receptors in the regulation of transcription have been repeatedly described (Liu et al., 1995; Chen et al., 1997; Cowley et al., 1997; Savatier et al., 2010; Cvoro et al., 2011; Tetel and Acharya, 2013). The energetics or affinity between ligands and receptors, between receptors, and between receptors and DNA can also be quantitatively related to transcriptional regulation (Agnati et al., 2007; Wang et al., 2013; Bain et al., 2014). A further regulatory mechanism of transcription is provided by epigenetic changes of the target genes’ HRS, namely DNA-methylation leading to reduced access of transcription factors to their binding sites and therefore, to reduced transcription of target genes. The opposite effect is provided by mechanisms of histone acetylation. Further, transrepression and transactivation of gene expression can be observed in steroid receptors (Newton and Holden, 2007), allowing a complex fine tuning of transcriptional activity. Pharmacological blockade of the ERα in young male rats for instance has significant effects not only on spatial performance but also on the expression of other steroid receptor genes (Meyer and Korz, 2013b).

The regulation of functional activity at the protein level is similarly manifold. The cellular distribution separates genomic functions mediated by cytosolic receptors from non-genomic effects mediated by membrane-bound receptors, although these receptors can indirectly be involved in the regulation of transcriptional activity via different cellular signaling processes (Norman et al., 2004). In addition, different subregions and cell types within the hippocampus may contribute differentially to receptor interactions. Colocalization of MR and GR has been described for CA1 and CA2 pyramidal and dentate gyrus granular neurons and nuclei in the rat brain (Han et al., 2005). ERα and ERβ are also widely colocalized in the the hippocampus (Hösli and Hösli, 1999), seemingly ERβ more in the cytoplasm and ERα more in the nuclei of CA1, CA3 and dentate gyrus neurons in male rats (Kalita et al., 2005).

A limitation of the study is the different age of animals, however regression analyses along three age classes gives very ambigious or even no results. There is no clear age specific delimitable concentration of animals in the figures making a age effect unlikely. However further studies should focus on separate analyses of age classes and should include females as well, in order to figure out sex specific differences.

Nevertheless, the obtained results make more detailed studies promising regarding the understanding of processes leading to the coordinated expression of steroid receptor genes and the interactions of receptors within a complex network to induce specific cellular signaling and target gene expressions during cognitive processes and stress related cognitive decline.

## Author Contributions

GL discussed the results, wrote the manuscript. VK generated the idea, performed the analysis, wrote the manuscript.

## Conflict of Interest Statement

The authors declare that the research was conducted in the absence of any commercial or financial relationships that could be construed as a potential conflict of interest. The reviewer AB and the handling Editor declared their shared affiliation, and the handling Editor states that the process nevertheless met the standards of a fair and objective review.
